# Putative roles as oncogene or tumour suppressor of the Mid-clustered microRNAs in *Gallid alphaherpesvirus 2* (GaHV2) induced Marek’s disease lymphomagenesis

**DOI:** 10.1099/jgv.0.000786

**Published:** 2017-05-11

**Authors:** Man Teng, Zu-Hua Yu, Pu Zhao, Guo-Qing Zhuang, Zi-Xiang Wu, Lu Dang, Hui-Zhen Li, Sheng-Ming Ma, Zhi-Zhong Cui, Gai-Ping Zhang, Run Wu, Jun Luo

**Affiliations:** ^1^​ College of Veterinary Medicine, Gansu Agricultural University, Lanzhou 730070, PR China; ^2^​ Key Laboratory of Animal Immunology of the Ministry of Agriculture, Henan Provincial Key Laboratory of Animal Immunology, Henan Academy of Agricultural Sciences, Zhengzhou 450002, PR China; ^3^​ College of Animal Science and Technology, Henan University of Science and Technology, Luoyang 471023, PR China; ^4^​ Department of Veterinary Pathobiology, College of Veterinary Medicine and Biomedical Sciences, Texas A&M University, College Station, TX, USA; ^5^​ College of Animal Science and Veterinary Medicine, Shandong Agricultural University, Taian 271018, PR China; ^6^​ College of Animal Science and Veterinary Medicine, Henan Agricultural University, Zhengzhou 450002, PR China; ^7^​ Jiangsu Co-innovation Center for Prevention and Control of Important Animal Infectious Diseases and Zoonoses, Yangzhou 225009, PR China

**Keywords:** Marek's disease virus, herpesvirus, GaHV2, miRNA, tumour suppressor, oncogene, miR-155

## Abstract

In the last decade, numerous microRNAs (miRNAs) have been identified in diverse virus families, particularly in herpesviruses. *Gallid alphaherpesvirus 2* (GaHV2) is a representative oncogenic alphaherpesvirus that induces rapid-onset T-cell lymphomas in its natural hosts, namely Marek’s disease (MD). In the GaHV2 genome there are 26 mature miRNAs derived from 14 precursors assembled into three clusters, namely the Meq-cluster, Mid-cluster and LAT-cluster. Several GaHV2 miRNAs, especially those in the Meq-cluster (e.g. miR-M4-5p), have been demonstrated to be critical in MD pathogenesis and/or tumorigenesis. Interestingly the downstream Mid-cluster is regulated and transcribed by the same promoter as the Meq-cluster in the latent phase of the infection, but the role of these Mid-clustered miRNAs in GaHV2 biology remains unclear. We have generated the deletion mutants of the Mid-cluster and of its associated individual miRNAs in GX0101 virus, a very virulent GaHV2 strain, and demonstrated that the Mid-clustered miRNAs are not essential for virus replication. Using GaHV2-infected chickens as an animal model, we found that, compared with parental GX0101 virus, the individual deletion of miR-M31 decreased the mortality and gross tumour incidence of infected chickens while the deletion individually of miR-M1 or miR-M11 unexpectedly increased viral pathogenicity or oncogenicity, similarly to the deletion of the entire Mid-cluster region. More importantly, our data further confirm that miR-M11-5p, the miR-M11-derived mature miRNA, targets the viral oncogene *meq* and suppresses its expression in GaHV2 infection. We report here that members of the Mid-clustered miRNAs, miR-M31-3p and miR-M11-5p, potentially act either as oncogene or tumour suppressor in MD lymphomagenesis.

## Abbreviations

BAC, bacterial artificial chromosome; CDS, coding sequence; CEF, chicken embryo fibroblast; DLRA, dual luciferase reporter assay; GaHV2, gallid alphaherpesvirus 2; MD, Marek's disease; MDV, Marek's disease virus; miRNA, microRNA; ncRNA, non-coding RNA; p.i., post-infection; primiR, primary miRNA; qRT-PCR, quantitative reverse-transcription PCR; RISC, RNA-induced silencing complex; UTR, untranslated region.

## Introduction

MicroRNAs (miRNAs) are a class of small non-coding RNAs (ncRNAs), about 22–24 nt long, which play important post-transcriptional regulatory functions by base pairing to form the RNA-induced silencing complex (RISC) and induce target mRNA degradation and/or translation inhibition affecting various biological processes such as development, differentiation, apoptosis, organ formation, metabolism and all aspects of cancer biology [1–3]. In the past decade, numerous miRNAs have been identified in mammals, plants and even in viruses [4]. Over 400 viral miRNAs have been identified, the vast majority encoded in various human or non-human herpesviruses, and some of these have been demonstrated to participate in viral pathogenesis and/or oncogenesis [5–8]. Avian herpesviruses include the pathogenic Marek’s disease virus type 1 (MDV-1) and the avirulent type 2 (MDV-2), recently reclassified as *Gallid alphaherpesvirus 2* (GaHV2) and *Gallid alphaherpesvirus 3* (GaHV3), respectively [9]. GaHV2 is a representative oncogenic alphaherpesvirus that induces rapid-onset T-cell lymphomas in its natural hosts, named Marek’s disease (MD), a neoplastic and immunosuppressive avian disease responsible worldwide for large financial losses in the poultry industry [10, 11]. The disease and GaHV2-induced tumours can be prevented by vaccination. As the first case in which cancers could be prevented by anti-viral vaccination, MD has historically been regarded as an ideal model for investigating the biology, genetics and immunology of viral tumorigenesis [12].

The life cycle of GaHV2 is complicated, and the course of MD has been well established as the ‘Cornell Model’ [13], which includes four stages: (a) the early cytolytic phase, (b) the latent phase, (c) the late-cytolytic and immunosuppressive phase and (d) the proliferative phase [14]. Although there is an extensive literature on MD biology during the course of disease, few of the molecular mechanisms of MD pathogenesis, particularly those mediated by viral genes, have been uncovered. In the ~180 kb long viral genomes of GaHV2 [15, 16], nearly 200 ORFs have been recognized but most are only characterized hypothetically as viral protein-coding genes. Furthermore, only the GaHV2-specific gene *meq* (MDV *EcoR*I-Q), encoding a basic leucine zipper protein, has been characterized as a major oncogene responsible for MD tumorigenesis [17]. The more recent studies of miRNAs in avian herpesviruses possibly provide new opportunities for revealing more fundamental molecular determinants that trigger the development of MD lymphomas.

**Fig. 1.**
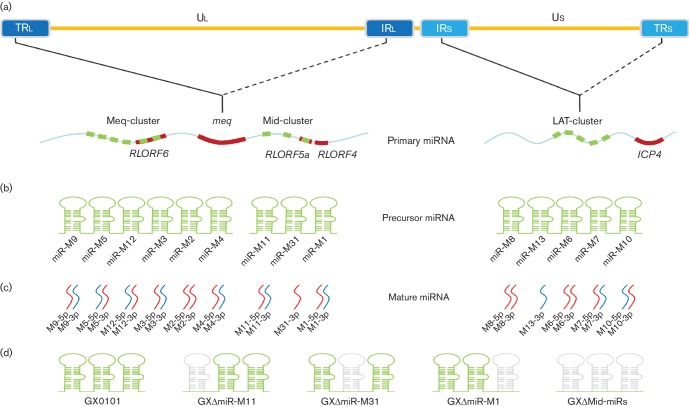

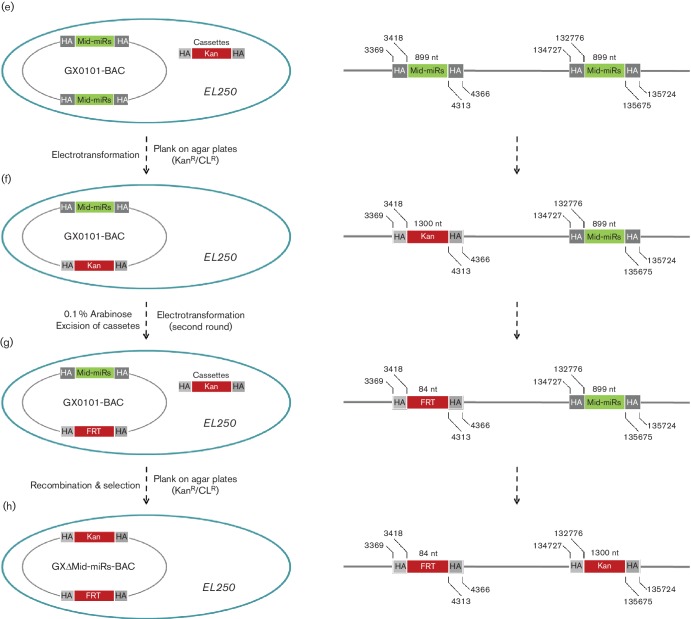
Schematics of GaHV2-encoded miRNAs and BAC mutagenesis strategy for miRNA deletion. (a) Primary transcripts of miRNAs. (b) miRNA precursors shown in hairpins. (c) Mature miRNAs shown as S line. (d) GaHV2 mutants with miRNA-deletions in the Mid- cluster. (e–h) Sequential steps for the deletion of the Mid-cluster by BAC mutagenesis. Relative locations of viral miRNAs at two identical genomic loci in inverted repeats ‘TR_L_/IR_L_’ and ‘IR_S_/TR_S_’ are shown by solid or dashed lines. The viral ORF and miRNA genes are indicated in bold by red or green curves, and miRNA precursors are shown as green hairpins while the mature miRNAs and passenger miRNAs are indicated by red or blue curved strands, respectively. The deleted miRNAs are shown as grey hairpins in the Mid-cluster of distinct GaHV2 mutants. The precursors and mature miRNAs are abbreviated, e.g. mdv1-miR-M4 and mdv1-miR-M4-5p are shortened to miR-M4 and M4-5p, respectively. Kan, kanamycin gene; CL, chloramphenicol gene; HA, 50-nt homology arms using for guiding the recombinations. The genomic loci are referenced to the viral genome of GaHV2 strain GX0101 (GenBank acc. no. JX844666). The sequential steps for the deletions of individual miRNAs in the Mid-cluster by BAC mutagenesis are demonstrated in Figs S1, S2 and S3 (available in the online Supplementary Material).

In GaHV2 genomes, as illustrated in Fig. 1(a–c), 26 mature miRNAs derived from 14 precursors are assembled into three clusters [18–20], namely the Meq-cluster (miR-M9 ~ miR-M4), the Mid-cluster (miR-M11 ~ miR-M1) and the LAT-cluster (miR-M8 ~ miR-M10), respectively. Recent advances have suggested that some of the GaHV2 miRNAs, especially those encoded in the Meq-cluster, are potentially critical for MD pathogenesis and/or oncogenesis [7]. Similarly to Kaposi’s sarcoma-associated herpesvirus (KSHV)-encoded miR-K12-11 [21], the most highly expressed miR-M4-5p encoded by GaHV2 has been characterized as a viral analogue of cellular miR-155 and specifically inhibits the translation of viral proteins involved in virus DNA cleavage/packaging [22, 23]. Since miR-155 is a host oncogene associated with several cancers [24, 25], miR-M4-5p has also been hypothesized to play a critical role in GaHV2 oncogenesis. Using a bacterial artificial chromosome (BAC) clone and Rec E/T recombination techniques, previous studies have shown that the deletion of miR-M4-5p from the viral genomes of GaHV2 strains with differing virulence can greatly reduce the oncogenicity of the virus [26, 27]. In a later study [28], we have shown that miR-M4-5p induces over-expression of the well-known oncogene *c-Myc* by targeting the latent TGF-β (transforming growth factor beta) binding protein 1 (LTBP1) and thus suppressing the TGF-β signalling pathway, providing further evidence for a direct *in vivo* role of a viral miRNA in MD lymphomagenesis. In addition, another miRNA, miR-M3-5p, was also found to be important for GaHV2 oncogenesis [29], by targeting and down-regulating SMAD2 expression, a critical component in the TGF-β signalling pathway that prolongs the longevity of infected cells. More recent studies have further revealed that as well as miR-M4-5p and miR-M3-5p, most deletions of the other individual miRNAs in the Meq-cluster will also significantly decrease the pathogenicity and oncogenicity of the virus [30], further supporting the close association of the Meq-clustered miRNAs to GaHV2 biology.

The Meq-cluster and the Mid-cluster of GaHV2 miRNAs are located upstream and downstream from the *meq* oncogene in IR_L_/TR_L_ regions (Fig. 1a, b) [18–20]. Interestingly, transcription of the two clusters gives two distinct transcriptional patterns at different phases of the infection [31]. During the latent phase, they are both driven by a single promoter, prmiRM9M4, while during the lytic phase they are transcribed separately. Compared with the critical significance of the Meq-clustered miRNAs that has been reported previously, the role of the Mid-clustered miRNAs in GaHV2 biology remains unclear. Herein, a series of GaHV2 mutants with deletions of the entire Mid-cluster or of the associated individual miRNAs have been reconstituted for animal experiments to enable evaluation of the Mid-clustered miRNAs and virus pathogenicity and/or oncogenicity. Furthermore, the candidate viral mRNA targets of the Mid-clustered miRNAs were simultaneously predicted and identified.

## Results

### Verification of BAC clones deleted in the Mid-clustered miRNAs from GX0101-BAC

**Fig. 2.**
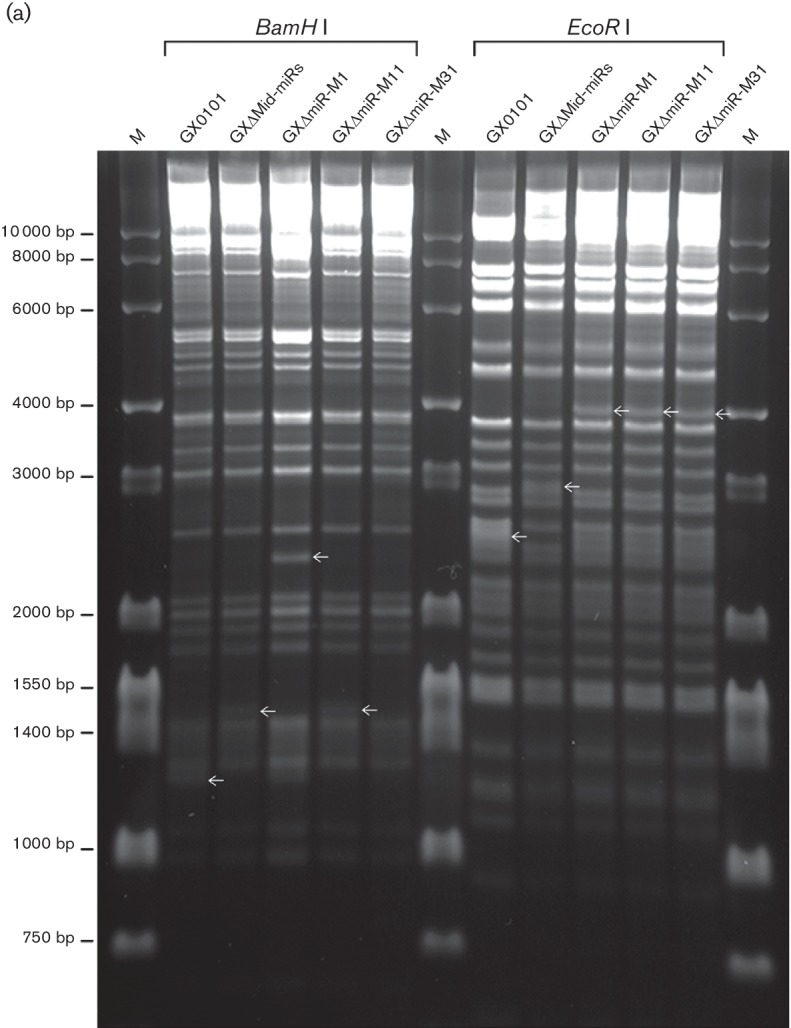

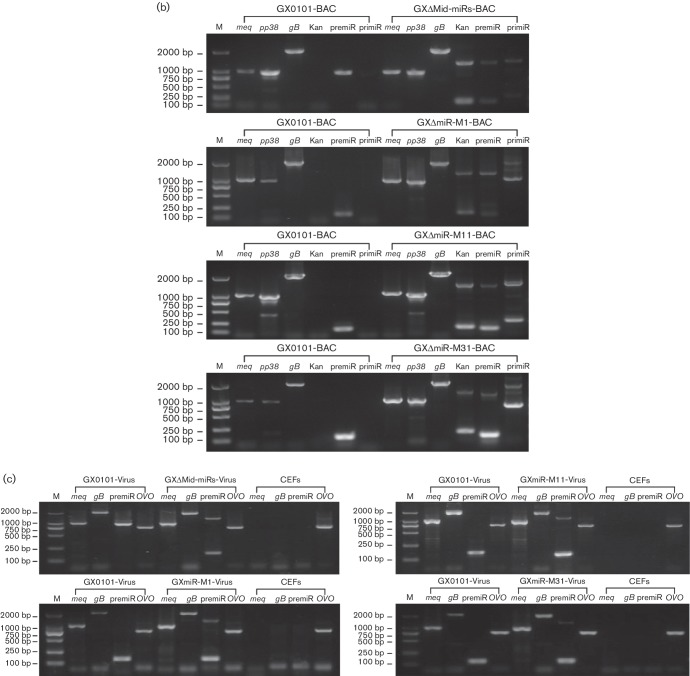
Identification of the reconstituted GaHV2 mutant with miRNA-deletions. (a) RFLP analysis of the integriity of GaHV2 genomes in BAC clones. The purified BAC plasmids of GX0101 or its mutants were digested by restriction enzymes *Bam*HI and *Eco*RI and were then analysed by electrophoresis on 1 % agarose gel. Bands changed as expected are shown by arrows. (b and c) Comparisons of the PCR products amplified from the BAC clones and reconstituted viral genomes of GaHV2 and its mutants. The BAC DNAs or corresponding viral genomic DNAs from miRNA-deleted mutants were used as templates. The parental GX0101 and chicken embryo fibroblasts (CEFs) serve as positive or negative controls, respectively. *m* *eq*, MDV *EcoR*I-Q gene; *pp38*, phosphorylated protein 38 gene; *gB*, glycoprotein B gene; Kan, kanamycin gene; premiR, miRNA precursor; primiR, primary miRNA; *OVO*, chicken ovotransferrin gene.

To evaluate the potential roles of the Mid-clustered miRNAs involved in GaHV2 oncogenesis, a series of BAC clones with corresponding deletions of the Mid-cluster and individual miRNA precursors including miR-M1, miR-M11 and miR-M31, as shown in Fig. 1(d–h), were firstly constructed by BAC mutagenesis based on a full-length infectious BAC clone of the very virulent GaHV2 strain GX0101 [32]. RFLP analysis, as shown in Fig. 2(a), was performed and demonstrated the integrity of GaHV2 genomes in BAC clones, with the changes as expected in corresponding miRNA-deleted genomic regions. Deletions of miRNAs were further verified by PCR analysis using purified plasmids of GX0101-BAC and GXΔmiR-BAC as templates, with specific primer pairs listed in Table S1. The agarose gel electrophoresis showed that identical products of all three representative GaHV2 genes, including *meq*, *pp38* and *gB*, were generated by PCR on all the five BAC DNA templates (Fig. 2b), demonstrating that the mutagenesis steps caused no effects on the replication of recombinant BACs. In comparison with GX0101-BAC, as demonstrated in Fig. 2(b), the presence of bands for the kanamycin (Kan) resistance gene and primary miRNA (primiR) genomic fragments indicate the chimeric genomic regions in GXΔmiR-BACs, whereas the changed bands of miRNA precursor (premiR) genomic fragments in GXΔmiR-BAC DNAs demonstrate the deletions of the Mid-clustered miRNA genes, which were further confirmed by DNA sequence analysis of the corresponding chimeric primiR genomic regions (Table S2).

### Characterization of GaHV2 mutants reconstituted from GXΔmiR-BACs

**Fig. 3.**
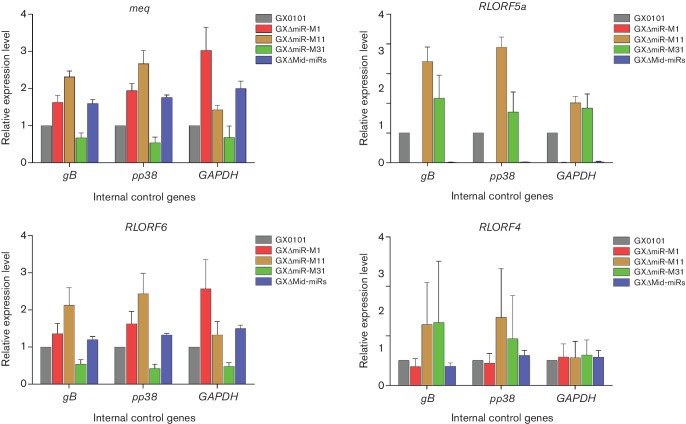
Relative expression levels of GaHV2 genes in CEFs infected with GX0101 or mutant viruses. Relative expression levels of the corresponding protein-coding genes were determined at 120 h post-infection. The GaHV2 genes *gB*, *pp38* and chicken *GAPDH* served as internal controls, respectively. The expression levels for the reference genes from the GX0101 group were set at 1. Error bars are derived from three replicates.

**Fig. 4.**
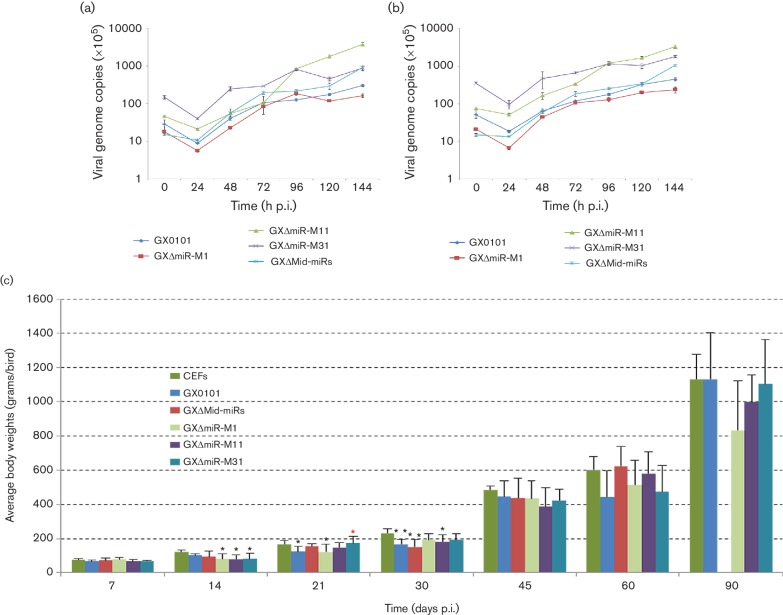

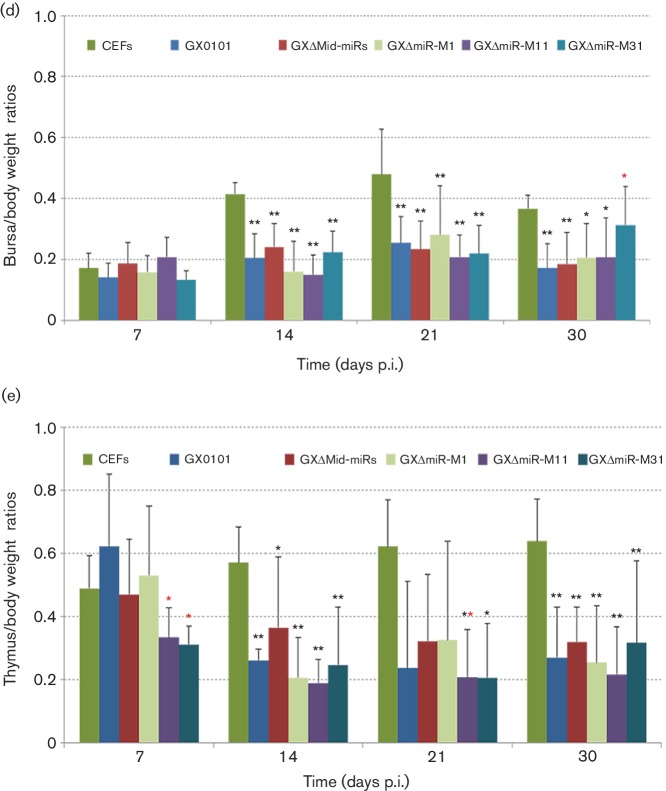
*In vitro* growth kinetics and *in vivo* immunosuppression effects on hosts of GX0101 and its mutants. *In vitro* growth kinetics of GX0101 and its mutants. Propagation rates of GX0101 and its engineered mutants measured as the viral genome copy numbers per 100 000 cells, based on GaHV2 *meq* and *gB* genes (a and b, respectively), were determined using real-time qPCR on DNA extracted from CEFs sampled at various time points after virus infection. (c, d and e) The body weights and the proportionate weights of bursa or thymus to body weight of birds following infection with GX0101 or its mutants. For each group, the body, bursa and thymus weights of six randomly selected surviving birds were measured at different time points post-infection (p.i.). The significant differences (*P*<0.05) from CEFs and GX0101 control groups are shown by black or red asterisks, respectively. For the body weight, no data is applicable for GX∆Mid-miRs at 90 days p.i. due to 100 % mortality by this time.

GaHV2 mutants with miRNA-deletion were reconstituted by transfection of BAC plasmids into chicken embryo fibroblast (CEF) confluent monolayers. Characteristic GaHV2 plaques were visible at about 1 week post-transfection. The viral plaques produced by all four of the reconstituted GXΔmiR and parental viruses were specifically stained using a gB-specific monoclonal antibody, and no distinguishable features were observed from each other (data not shown). When using viral genomic DNA as templates, as demonstrated in Fig. 2(c) and Table S2, confirmation of the deletions of corresponding Mid-clustered miRNAs from the viral genomes of the reconstituted GXΔMid-miRs, GXΔmiR-M1, GXΔmiR-M11 and GXΔmiR-M31 viruses was found by both PCR analysis of reconstituted viral genomic DNAs and subsequent sequencing. A quantitative reverse-transcription PCR (qRT-PCR) analysis showed that except for the undetectable deleted miRNAs, both the miR-M4-5p and neighbour miRNAs in the Mid-cluster were normally expressed in CEFs infected with GX0101 or mutants with associated miRNA-deletions (data not shown). The results further confirmed the successful deletions of corresponding Mid-clustered miRNAs from viral genomes of the reconstituted viruses. Furthermore, expression levels of the GaHV2 protein-coding genes neighbouring the Mid-cluster were simultaneously analysed by qRT-PCR. As demonstrated in Fig. 3, miRNA-deletions had no effects on the normal expression levels of *meq*, *RLORF6*, *RLORF5a* and *RLORF4* in any of the mutant virus-infected CEFs, except for the undetectable *RLORF5a* as expected in GXΔmiR-M1- and GXΔMid-miRs-infected CEFs. In addition, the *in vitro* virus proliferation curves were determined by a quantitative real-time PCR assay and showed that in GaHV2-infected CEFs, both the parental GX0101 and its mutants showed very similar replication kinetics (Fig. 4a, b). This implies that these Mid-clustered miRNAs are not essential for GaHV2 replication.

### Growth rates of birds infected with miRNA-deleted GaHV2 mutants

To compare the pathogenicity of both the parental GX0101 virus and its mutants, we conducted animal experiments and examined the growth rates of infected birds. For each group, consisting of 76 one-day-old chickens, the birds were challenged separately with GX0101 or mutant viruses while the negative controls were inoculated with an equal volume of mock CEFs. In all six experimental groups, as shown in Fig. 4(c), no difference in body weight of birds was observed in the first 7 days post-challenge. GX0101 strongly inhibited the growth rates of infected birds only between 21 and 30 days, after which the surviving birds appeared to grow normally in the last two months of the experimental time period. The miRNA-deletion mutants showed a similar effect on the birds' growth.

### Influence of the Mid-cluster miRNA deletions on chicken immune organs

For the first week post-challenge, as demonstrated in Fig. 4(d, e), no change in the bursa/body weight or thymus/body weight ratios of the virus-infected birds was observed. From 14 to 30 days there was in general a significant fall in the ratios of both bursa/body weight and thymus/body weight. For the bursa/body weight ratios, those of birds challenged by the parental GX0101 or any of the mutant viruses were significantly lower (*P*<0.05) than those of the mock controls (Fig. 4d). For most cases during this time period, no significant difference between GX0101 and its mutants was recorded in bursal weight ratio, except for the GXΔmiR-M31-infected birds measured at 30 days post-infection (p.i.). Similarly, except for the time point of 21 days p.i., no difference in the thymus/body weight ratios between birds challenged by the parental GX0101 or its mutants was observed although all groups of infected birds showed an obvious fall in thymus/body weight ratio compared with the mock control group (Fig. 4e).

### Pathogenicities of parental GX0101 and the derivative miRNA-deleted viruses

**Fig. 5.**
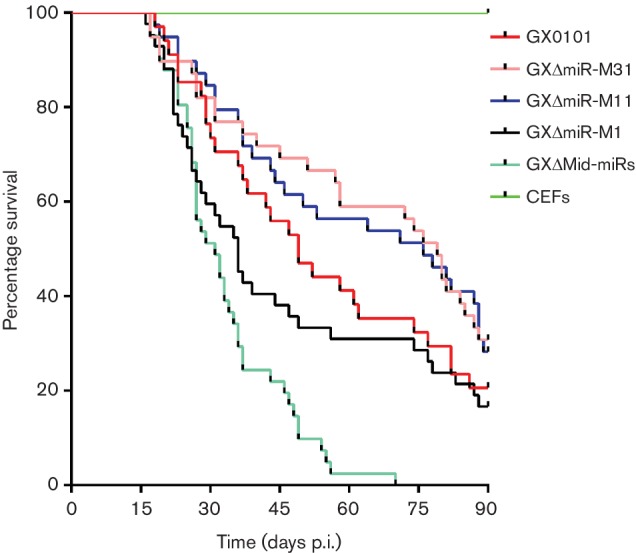
Survival curves of the birds challenged with GX0101 and its mutants during the 90-day experimental time period. For each of the five experimental groups, one-day-old birds were infected separately with CEFs containing 2000 p.f.u. of each variant GaHV2 virus by abdominal cavity inoculation. A sixth group of birds was inoculated with an equal volume of mock CEFs to serve as negative controls. The data are calculated using GraphPad Prism version 6.0.

For animal experiments, each of 76 birds was used for virus challenge. As shown in Table S3, the birds randomly selected and sacrificed for sample collection and those suffering early death before 14 days p.i. were removed, and then the remaining birds were observed for calculating the mortality and gross tumour occurrence. Post-challenge, as shown in Table 1, birds from the GX0101-infected group developed MD rapidly, some of which showed classic clinical nervous symptoms, and mortality started as early as 21 days p.i. and continued at an increasing rate until by 90 days p.i. it had reached 79.4 %. No death was recorded in birds from the mock control group. By comparison, birds inoculated with the miRNA-deleted mutants showed very differential courses of disease. As demonstrated in Fig. 5 and Table 1, the viruses with miRNAs deleted individually, GXΔmiR-M31, GXΔmiR-M11 and GXΔmiR-M1, gave cumulative mortalities of 69.2, 71.8 and 83.3 %, respectively, at the end of the 90-day experimental time period. However, infection with the virus having the entire Mid-cluster deleted, GXΔMid-miRs, unexpectedly gave the strongest and most significant (*P*<0.01) response, resulting in a 100 % mortality in infected birds. The compatible cumulative gross tumour incidences by the end of 90 days recorded in birds infected with GX0101, GXΔmiR-M31, GXΔmiR-M11 or GXΔmiR-M1, as shown in Table 1, were 41.2, 41.0, 46.2 and 28.6 %, respectively. The significance of the difference in mortality and gross tumour occurrence in birds compared with each of the viruses is shown in Table S4. Furthermore, once the sample size was enlarged and included the birds that were sacrificed at time points of 21, 30, 45 and 60 days p.i., totals of 40.0, 31.7, 45.0 and 31.7 % of birds were found to have gross tumour occurrences in birds infected with GX0101, GXΔmiR-M31, GXΔmiR-M11 or GXΔmiR-M1 virus, respectively (Table S5).

**Table 1.** Cumulative death and mortality in MDV-challenged chickens calculated at different time points (days p.i.)

Time point (days p.i.)	Category	MDV strains and mock group*					
		GX0101 (34)	GX∆miR-M31 (39)	GX∆miR-M11 (39)	GX∆miR-M1 (42)	GX∆Mid-miRs (41)	Mock CEFs (40)
15	Deaths	–	–	–	–	–	–
	Mortality	–	–	–	–	–	–
	Gross tumours	–	–	–	–	–	–
	Tumour incidence	–	–	–	–	–	–
30	Deaths	9	7	6	17	20	–
	Mortality	26.5 %	17.9 %	15.4 %	40.5 %	48.8 %	–
	Gross tumours	5	2	2	3	4	–
	Tumour incidence	14.7 %	5.1 %	5.1 %	7.1 %	9.8 %	–
45	Deaths	15	12	14	26	32	–
	Mortality	44.1 %	30.8 %	35.9 %	61.9 %	78.0 %	–
	Gross tumours	6	3	6	3	6	–
	Tumour incidence	17.6 %	7.7 %	15.4 %	7.1 %	14.6 %	–
60	Deaths	20	16	17	29	40	–
	Mortality	58.8 %	35.9 %	43.6 %	69.0 %	97.6 %	–
	Gross tumours	6	4	8	4	7	–
	Tumour incidence	17.6 %	10.3 %	20.5 %	9.5 %	17.1 %	–
75	Deaths	23	18	19	30	41	–
	Mortality	67.6 %	46.2 %	48.7 %	71.4 %	100.0 %	–
	Gross tumours	8	5	10	5	7	–
	Tumour incidence	23.5 %	12.8 %	25.6 %	11.9 %	17.1 %	–
90	Deaths	27	27	28	35	/	–
	Mortality	79.4 %	69.2 %	71.8 %	83.3 %	/	–
	Gross tumours	14	16	18	12	/	–
	Tumour incidence	41.2 %	41.0 %	46.2 %	28.6 %	/	–

–, No death or gross tumour caused by virus infection was observed at the corresponding time points post-challenge.

/, There is no data applicable due to the death of all GX∆Mid-miRs-challenged chickens before 75 days p.i.

*Not including the birds sacrificed at 7, 10, 14, 21, 30, 45 and 60 days p.i. for sample collections and early deaths occurring before 14 days p.i. possibly due to intraperitoneal infection, totals of 34, 39, 39, 42, 41 and 40 birds remained in the GX0101, GX∆miR-M31, GX∆miR-M11, GX∆miR-M1, GX∆Mid-miRs and mock CEFs groups and these were observed for calculating the deaths/mortality and gross tumour occurrence, respectively.

### Prediction and characterization of viral mRNA targets of the Mid-clustered miRNAs

**Fig. 6.**
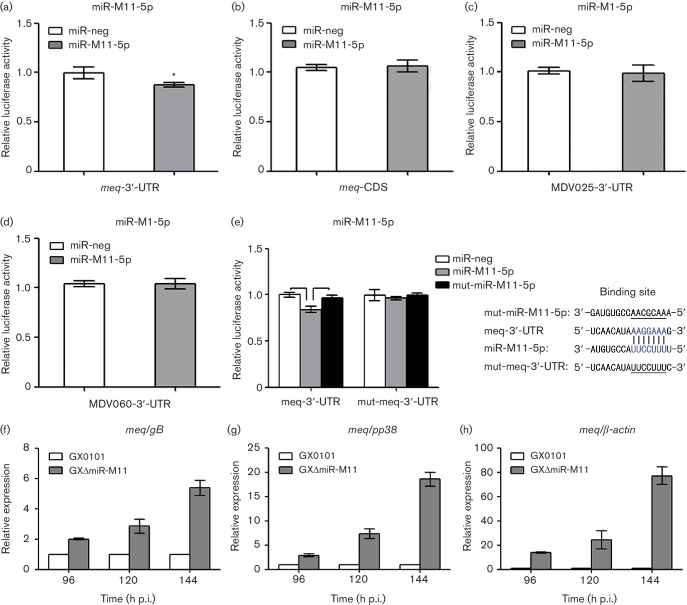
Interactions between the Mid-clustered miRNAs and candidate viral mRNA targets. (a–e) Dual luciferase reporter assay analysis of the interactions between miR-M11-5p, miR-M1-5p and 3′-UTRs or CDS of *meq*, *MDV025* and *MDV060* genes. The columns show the means of *Renilla*-to-firefly luminescence ratios. Error bars are derived from three independent replicates. Where the difference gives *P*<0.05, this is indicated as single asterisk. On the right in (e), the miRNA/mRNA binding site and corresponding mutants in miR-M11-5p and the 3′-UTR of the *meq* gene are shown in blue or underlined. (f–h) Relative expression levels of the *meq* gene in CEFs infected with GX0101 or GX∆-miR-M11 viruses. The GaHV2 genes *gB* and *pp38* and chicken *β-actin* serve as internal controls, respectively. The expression levels for the reference genes from the GX0101 group were set at 1. Error bars are derived from three replicates.

To reveal potential mechanisms mediated by the Mid-clustered miRNAs, prediction of possible viral mRNA targets of all five miRNAs encoded in the Mid-cluster were sought by a bioinformatics approach utilizing ‘RNAhybrid’. A total of three candidate targets specific for miR-M1-5p or miR-M11-5p were obtained from the viral genome of GaHV2. As shown in Table S6, each of the three candidates *MDV025*, *MDV060* and the oncogene *meq* contains one potential binding site in the 3′ untranslated region (3′-UTR), together with two predicted sites in the coding sequence (CDS) of the *meq* gene. To find out whether the two viral miRNAs bind directly to the five predicted sites in 3′-UTR or CDS of *MDV025*, *MDV060* and/or *meq*, we performed a site-directed dual luciferase reporter assay (DLRA). For the first round of the DLRA, as demonstrated in Fig. 6(a–d), only the *meq*-3′-UTR reporters were significantly repressed by miR-M11-5p, compared with the negative control miR-neg (*P*<0.05). To ensure that this observation represented the seed-sequence-dependent down-regulation of the target by miR-M11-5p, a specific mutant vector of miR-M11-5p, as shown on the right-hand side in Fig. 6(e), was constructed. Then, the second round of the DLRA was performed and showed that when the seed sequence of miR-M11-5p was mutated, the repression effect on reporters was lost (Fig. 6e, black column on the left). Conversely the specific repression by miR-M11-5p on the *meq*-3′-UTR reporter depended on the predicted binding site, which was confirmed by the generation of mutant *meq*-3′-UTR reporters and the performance of reporter assays as above. As expected, the mutant binding site lost its response to miR-M11-5p (Fig. 6e, grey column on the right), indicating the specificity of the repression.

### miR-M11-5p down-regulates mRNA expression of the viral oncogene *meq* in infected CEFs

To demonstrate *in vivo* whether the miRNA/mRNA target interaction occurred, CEFs were infected with either the parental virus GX0101 or its mutant GXΔmiR-M11. The characteristic GaHV2 plaques were clearly visible at 96 h p.i., and had enlarged and spread rapidly to adjacent cells at 120 and 144 h p.i. Cultures were collected at these three time points for subsequent qRT-PCR analysis. As shown in Fig. 6(f–h), the mRNA expression level of *meq* was obviously up-regulated in GXΔmiR-M11-infected CEFs at every time point, compared with that in GX0101-infected CEFs. This further indicates that the GaHV2 oncogene *meq* is a biological target of miR-M11-5p.

## Discussion

miRNAs have been shown to play important post-transcriptional regulatory functions in multiple cellular processes. Earlier studies of the oncogenic GaHV2 have demonstrated that most of the Meq-clustered miRNAs, especially miR-M4-5p, a virus-encoded miR-155 analogue, play critical roles in MD lymphomagenesis [22, 23, 26–30]. The Meq-clustered miRNAs are located upstream in the viral oncogene *meq* while the Mid-clustered miRNAs lie downstream [20]. To reveal possible biological roles of the downstream Mid-clustered miRNAs, we constructed a series of GaHV2 mutants utilizing BAC mutagenesis of an infectious BAC clone derived from the very virulent GaHV2 strain GX0101 with deletions of the whole Mid-cluster or each of the individual miRNAs. Our data showed that all four of the miRNA-deleted mutants, including GXΔmiR-M1, GXΔmiR-M11, GXΔmiR-M31 and GXΔMid-miRs, gave similar replication kinetics in CEFs compared with the parent virus GX0101. This implies that the Mid-clustered miRNAs are not essential for virus replication, similar to the Meq-clustered miRNAs [26, 27, 30].

Compared with the parental virus GX0101, mutant viruses with deletions of any of the three individual miRNAs or the Mid-cluster produced a reduction in the weights of both the bursa of Fabricius and the thymus similar to that seen with the Meq-clustered miRNAs [27, 30], suggesting that these miRNAs are not directly involved in regulating the damage to immune organs. However, differentially from that of the Meq-clustered miRNAs, the body weight of birds infected with the mutants of the Mid-clustered miRNAs was inhibited similarly to those with GX0101 up to 30 days after infection. More importantly, deletions of the distinct Mid-clustered miRNAs variably changed the pathogenicity and/or oncogenicity of the virus during the whole experimental time period of 90 days. Compared with the parental GX0101 virus, the cumulative mortality and gross tumour incidence in birds infected with the mutant virus GXΔmiR-M31 were both reduced, similar to that observed with the Meq-clustered miRNAs [27, 30]. Previously, the miR-M31-derived mature miRNA miR-M31-3p has been shown to share a conserved seed sequence with miR-221 [33], which targets and suppresses a key cell cycle inhibitory regulator p27^Kip1^ protein, together with miR-222, in the induction and progression of T-cell lymphomas [34]. This implies that miRNAs derived from miR-M31 may act as potential oncogenes, likely to some of the Meq-clustered miRNAs [26, 27, 30].

However, compared with the parental GX0101 virus, mortality of the mutant virus GXΔmiR-M1-infected birds was slightly increased from 79.4 to 83.3 %. As for the oncogenicity, a similar phenomenon was also observed in GXΔmiR-M11-infectect birds, of which the cumulative gross tumour incidence at 90 days p.i. at the end of the animal experiment was increased from 41.2  to 46.2 %. This is quite different from that observed with the Meq-clustered miRNAs [27, 30] and suggests that the other Mid-clustered miRNAs are also critical for GaHV2 pathogenesis but possibly exert conversely regulatory roles in the induction of MD lymphomas. Although the deletion of miR-M1 interrupted the overlapping *RLORF5a* gene, it has been previously demonstrated that *RLORF5a* is dispensable for GaHV2 replication and oncogenicity [35, 36]. In previous work [37, 38], we have demonstrated that compared with most of the highly expressed Meq-clustered miRNAs, most of the Mid-clustered miRNAs are expressed at lower levels during the course of disease. The expression of miR-M11-5p derived from miR-M11 was nearly undetectable by Northern blotting [37]. However, we have recently performed a scan to look for possible candidate viral mRNA targets of the Mid-clustered miRNAs and identified the major GaHV2 oncogene *meq* as an *in vivo* biological target for miR-M11-5p. Combining previous reports and our present data on the associations of individual Mid-clustered miRNAs with virus pathogenicity and/or oncogenicity, we suggest that during GaHV2 infection miR-M31-3p possibly acts as an oncogene contributing to MD tumorigenesis while miR-M11-5p may act as a tumour suppressor.

**Fig. 7.**
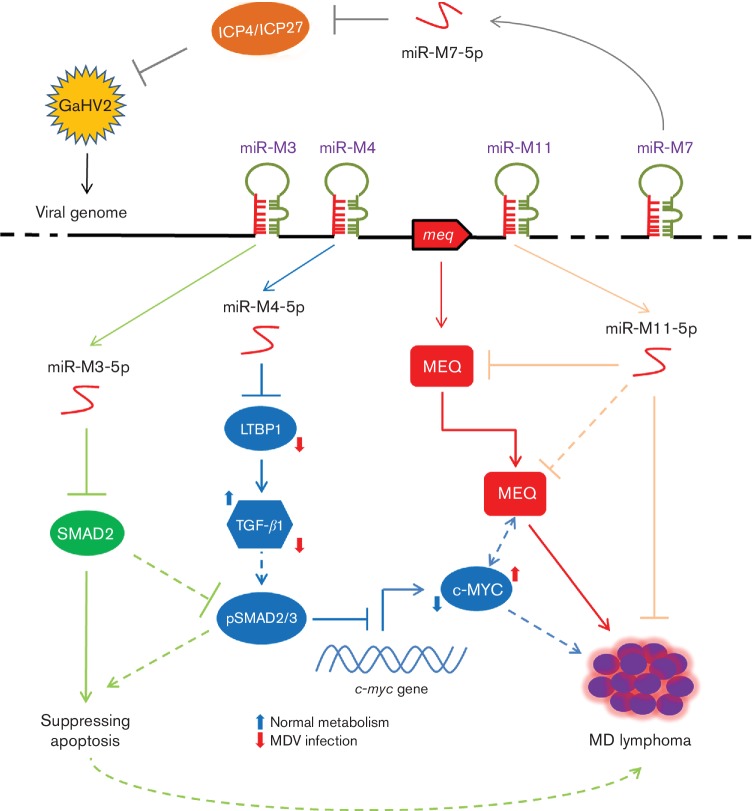
Schematic of a suggested primary regulatory network mediated by viral miRNAs in GaHV2 infection. The precursor and mature miRNA are shown by green hairpins or red S lines, respectively. The confirmed or suggested regulatory mechanisms of each miRNA are shown in different colours. Arrows and T-shaped lines indicate the *cis*-acting regulatory or suppression effects, respectively.

It is interesting that compared with the Meq-clustered miRNAs that contribute much to GaHV2 pathogenicity and oncogenesis [26, 27, 30], deletion of the whole Mid-cluster, mutant virus GXΔMid-miRs, significantly decreased virus pathogenicity and resulted in a 100 % mortality in infected birds. This may be explained by a suggestion that the co-operation of miR-M1 and miR-M11 may act as more important suppressors in the development of the disease, in contrast to a promotion by miR-M31. The GaHV2 miRNAs encoded in distinct gene clusters are all important potential regulators but possibly exert contrasting roles in the induction of MD lymphomagenesis. As demonstrated in Fig. 7, the present known mechanisms mediated by viral miRNAs co-operating with the *meq* gene in GaHV2 biology are presented. Amongst the Meq-clustered miRNAs, miR-M4-5p had been characterized as an oncogenic miRNA that targets multiple viral and host mRNAs to trigger MD lymphomagenesis [22, 23, 28, 39]. MiR-M3-5p, another miRNA encoded in the Meq-cluster, was also demonstrated to contribute to MD oncogenesis by targeting SMAD2 [29], an important component of the TGF-β signalling pathway. This pathway is also down-regulated by miR-M4-5p through targeting LTBP1 and finally activating the over-expression of *c-Myc* [28]. Previously, the MEQ protein has been characterized as a major oncoprotein [17] and has been proposed to form heterodimers with c-Myc [12]. The co-operation of the viral oncogene and miRNAs may be highly efficient triggers for the induction of MD lymphomagenesis. However, this process may be partly blocked by miR-M11-5p (Fig. 7), which is one of the Mid-clustered miRNAs downstream of the *meq* gene and has been presently characterized as a putative tumour suppressor targeting and down-regulation of expression of *meq*. A good explanation for this may be that the transcription of the Meq- and Mid-clusters has two distinct transcriptional patterns during distinct phases of GaHV2 infection [31]. During the latent phase, they are driven by a single promoter, prmiRM9M4, while during the lytic phase they are transcribed separately using independent promoters. The co-operation between viral miRNAs in the Meq- or Mid-clusters could be an advantage to the virus for establishing, maintaining latency and/or triggering tumorigenesis.

Furthermore, miR-M7-5p, one of the LAT-clustered miRNAs, has been verified to target the immediate-early genes ICP4 and ICP27 (Fig. 7), suggesting a role contributing to establishment and/or maintainance latency [40]. In the three miRNA gene clusters, GaHV2 encodes a total of 26 miRNAs but the biological functions of most miRNAs still remain unclear. Utilizing the BAC mutagenesis we have recently constructed a series of mutants of the Mid-clustered miRNAs and preliminarily investigated the effects on virus pathogenicity and oncogenicity without associated revertants due to the limited number of available isolators. Although the RFLP analysis of the recombinants and sequencing of the chimeric primiR genomic regions confirmed that no major mutations occurred in the BACs and that these mutants do not appear to be defective in lytic replication, the revertants would be needed to exclude possible mutations elsewhere in the BACs and mutated viral genomes if we focus on their individual functions in future work. Herein, our work has provided an important basis for further revealing the molecular regulatory mechanisms mediated by the Mid-clustered miRNAs in MD lymphomagenesis. Along with the reverse genetic manipulation techniques and miRNA target identification strategies and their application in research on herpesviruses, it is expected that more details of the regulatory network for miRNAs in herpesvirus biology will naturally follow.

## Methods

### Ethics statement

All experimental protocols were approved by the Laboratory Animal Management Committee of Key Laboratory of Animal Immunology, Ministry of Agriculture, China. Animal experiments with chickens were conducted following the protocols of the Laboratory Animal Management Committee of Key Laboratory of Animal Immunology, Ministry of Agriculture, China, which approved the permit (no. 2007001).

### Virus and cells

An infectious BAC-derived GX0101 virus, retaining its very virulent pathogenicity of GaHV2 [32], was used as a parental virus for the construction of GaHV2 mutants with deletions of the Mid-cluster or associated individual miRNAs. Primary CEF monolayers were prepared from nine-day-old specific-pathogen-free embryos (Jinan Spirax Ferrer Poultry Science and Technology) for virus propagation, and the viral titration by p.f.u. was measured as described previously [37, 41]. 293T cells (American Type Culture Collection) were cultured in DMEM (Dulbecco’s modified Eagle’s medium) supplemented with 10 % FBS (Gibco). All cells were maintained at 37 °C in a 5 % CO_2_ incubator.

### Construction of miRNA-deleted BAC clones

Construction of a series of the Mid-cluster or individual Mid-clustered miRNA-deleted GX0101-BAC clones, namely GXΔMid-miRs-BAC, GXΔmiR-M1-BAC, GXΔmiR-M11-BAC and GXΔmiR-M31-BAC, respectively, were made as described previously [27, 30]. Briefly, as demonstrated in Figs 1(e–h), S1, S2 and S3, *Escherichia coli* EL250 cells transformed with a GX0101-BAC containing the whole genome of GX0101 were prepared by inoculating a fresh overnight culture into 10 ml of Luria–Bertani (LB) medium containing chloramphenicol (25 µg ml^−1^) until an optical density at 600 nm of 0.5 was reached. Expression of *recE*, *recT* and *λ gam* was then induced by incubating at 42 °C for 15 min, and cells were collected for the preparation of electrocompetent cells by a standard protocol [42, 43]. Kan^R^ cassettes flanked by FRT sites were amplified using primers MidmiRF-Kan^R^, MidmiRR-Kan^R^, miR1F-Kan^R^, miR1R-Kan^R^, miR11F-Kan^R^, miR11R-Kan^R^, miR31F-Kan^R^ and miR31R-Kan^R^ (primer pairs 1−4, Table S1) from pKD13 [44]. After digesting with *Dpn*I to remove the residual pKD13 template, the PCR products were electrophoresed and purified using a Gel Extraction Kit (OMEGA). About 300 ng of the PCR products was electroporated into 50 µl of electrocompetent EL250 cells harbouring the GX0101-BAC using standard electroporation parameters (2.0 KV, 200 Ω and 25 µF). After electroporation, the cells were grown in 1 ml SOC medium (Sigma) for 2 h and spread onto LB agar plates containing chloramphenicol (25 µg ml^−1^) and kanamycin (50 µg ml^−1^). Resistant colonies were picked and grown in liquid LB medium. Excision of the Kan^R^ cassettes was carried out by induction of FLPe recombinase by adding 0.1 % arabinose into the medium. To delete the second miRNA allele from the TR_L_ or IR_L_ regions, another round of BAC mutagenesis was performed in the same way, except for the excision of the secondly recombined Kan^R^ cassettes. Finally, the EL250 cells harbouring GXΔMid-miRs-BAC, GXΔmiR-M1-BAC, GXΔmiR-M11-BAC or GXΔmiR-M31-BAC were grown up and the BAC DNA was prepared using Plasmid Midi Kits (QIAGEN) according to the manufacturer’s instructions.

### Confirmation of the deletion of miRNAs from GXΔmiR-BACs

Utilizing the restriction enzymes *Bam*HI and *Eco*RI (New England BioLabs), the RFLP analysis was first performed to investigate the integrity of GaHV2 viral genomes in the miRNA-deleted BAC clones. The deletion of the Mid-cluster or of individual Mid-clustered miRNA from the reconstituted BACs was then further analysed and confirmed by PCR amplification, utilizing the primer pairs listed in Table S1 that were designed for specific viral genomic regions or genes (primer pairs 5−15), the kanamycin resistance gene (primer pair 16) and the chicken ovotransferrin gene (primer pair 17), respectively. For final confirmation, all corresponding PCR products covering the Mid-clustered miRNAs were cloned conventionally and sent for DNA sequencing by Sangon Biotech (Shanghai, China).

### Reconstitution and confirmation of miRNA-deleted virueses

The BAC DNA of each miRNA mutant was prepared and transfected separately into CEF monolayers to rescue the corresponding reconstituted virus as previously described [27, 30]. Indirect immunofluorescence assay was performed for the characterization of rescued viruses as described previously [32]. After several rounds of passage to enrich the viral titres, GXΔMid-miRs, GXΔmiR-M1, GXΔmiR-M11 or GXΔmiR-M31 virus stocks were stored in liquid nitrogen. Similarly to that of the reconstituted BAC clones, confirmation of the deletions of miRNAs from the viral genomes was performed by PCR amplification and DNA sequencing.

### Determination of *in vitro* proliferation of GaHV2 mutants

The virus titres, as number of p.f.u., of both GX0101 and mutant strains were measured using CEF monolayers in 96-well plates as described previously [37]. CEF monolayers on 6-well plates were infected separately with 100 p.f.u. of a distinct GaHV2 strain and sampled at 0, 24, 48, 72, 96, 120 and 144 h p.i. Total DNAs were extracted, and the determination of the *in vitro* virus proliferation rates was performed by real-time quantitative PCR (qPCR) assays as described previously [27, 30], using the specific primers for GaHV2 *meq* and *gB* and chicken ovotransferrin (OVO) genes (primer pairs 18–20, Table S1).

### Animal experiments

The animal experiments with chickens were conducted according to the local protocols of the Ethical and Animal Welfare Committee of Key Laboratory of Animal Immunology, Ministry of Agriculture of China. A total of five experimental groups, each with 76 one-day-old white Leghorn specific-pathogen-free chickens (Jinan Spirax Ferrer Poultry Science and Technology), were separately challenged with CEFs containing 2000 p.f.u. of GX0101, GXΔMid-miRs, GXΔmiR-M1, GXΔmiR-M11 or GXΔmiR-M31 viruses by abdominal cavity inoculation. An additional group of 76 birds was simultaneously inoculated with equal doses of CEF cultures and served as the mock control. The birds were bred separately in isolators with filtered air under positive pressure in the animal facility. Post-challenge, birds were inspected regularly for any clinical symptoms and mortality. The procedure and number of birds randomly selected for sample collection are shown in Table S3. At the end of 90 days, all surviving birds were humanely euthanized and their organs examined for lesions at necropsy. Excluding the birds sacrificed for sample collection and those suffering early death before 14 days p.i. possibly due to the adverse effects of intraperitoneal infection, the rates of cumulative mortality and tumour generation were used to evaluate the pathogenicity and oncogenicity of the virus mutants. The significant differences in the mortality and gross tumour occurrence in birds of each group were calculated using GraphPad Prism version 6.0 (GraphPad Software).

### Evaluation of body and immune organ weights of birds

To evaluate the effects of GX0101 and its mutants on the birds' growth, six birds were randomly selected from each group and their body weights were measured at 7, 14, 21, 30, 45, 60 and 90 days p.i. For determining the immune organ weight, six birds from each group were simultaneously sacrificed on days 7, 14, 21 and 30 post-challenge and their bursa and thymus collected and weighed. Statistically, the differences in body weight and immune organ weight between groups challenged with different GaHV2 strains were compared and analysed by one-way analysis of variance (Oneway ANOVA, LSD) and were considered significant at a probability level of *P*<0.05.

### Bioinformatics prediction of miRNA targets

The protein-coding genes known or hypothesized in the viral genome of the Md5 strain (GenBank acc. no. AF243438) were used for the prediction of viral mRNA targets of the Mid-clustered miRNAs, utilizing the online bio-software ‘RNAhybrid’ [45]. For target screening, the criteria were set as follows: a perfect Watson–Crick match of miRNA/mRNA at positions 2–7 of miRNA and disallowance of G:U pairs in the ‘seed’.

### Vector construction for expression of miRNA and target sequence

A series of vectors containing miRNA precursor or viral gene containing predicted target site were first constructed for primary screening of viral mRNA targets. The viral genomic DNAs and CEF cellular RNAs were extracted from GX0101-infected CEFs as described previously [27, 30]. The precursor genes of miR-M1 and miR-M11 were amplified using primer pairs 1 and 2 (Table S7) and then cloned into the *Xho*I and *Not*I sites of plasmid pcDNA6.2 (Invitrogen) to construct the vectors pcDNA6.2-miR-M1-5p and pcDNA6.2-miR-M11-5p, respectively. Reverse transcription PCR (RT-PCR) was performed to amplify the wild-type 3′-UTR and CDS of the *meq* gene using primer pairs 3 and 4 (Table S7). The PCR products of *meq* and annealed 3′-UTRs of *MDV025* and *MDV060* (primer pairs 5 and 6, Table S7) were purified and cloned into the *Eco*RI and *Xho*I sites of psiCHECK-2 (Promega) to construct four vectors, namely psiCHECK-2-meq-3′-UTR, psiCHECK-2-meq-CDS, psiCHECK-2-MDV025-3′ -UTR and psiCHECK-2-MDV060-3′-UTR, respectively. Mutants of miR-M11 precursor and partial meq-3′-UTRs were obtained by annealing the oligonucleotides (primer pairs 7 and 8, Table S7) and then similarly cloned into psiCHECK-2 to construct the corresponding vectors named pcDNA6.2-mut-miR-M11-5p and psiCHECK-2-mut-meq-3′-UTR, respectively. Primers and oligonucleotides listed in Table S7 were synthesized by Sangon Biotech (Shanghai, China).

### Dual luciferase reporter assay

The determination of miRNA/mRNA interactions was performed by DLRA. Briefly, the mixed plasmids, psiCHECK-2 vector containing each 3′-UTR, CDS or their mutants plus vectors pcDNA6.2-miR-M1-5p, pcDNA6.2-miR-M11-5p, pcDNA6.2-mut-miR-M11-5p or pcDNA6.2-miR-neg were co-transfected into the confluent 293T cells, maintained in a 5 % CO_2_ incubator at 37 °C for 48 h and then analysed using the dual-luciferase reporter system as described previously [28]. Both firefly luciferase activity and *Renilla* luciferase activity were determined using a GloMax 96 Microplate Luminometer (Promega). Each experiment and determination was repeated independently in triplicate, and then the data were calculated as means ± standard deviations (sd) utilizing the software GraphPad Prism (version 6.0).

### Virus infection of CEFs with GaHV2

The confluent CEF cultures in 6-well plates were infected with GX0101 or its mutant viruses (each of 100 p.f.u. per well) and then maintained in DMEM medium supplemented with 1 % FBS (Gibco) at 37 °C in a 5 % CO_2_ incubator. At 96, 120 and 144 h p.i., the cell cultures were collected for subsequent qRT-PCR analysis.

### Quantitative reverse-transcription PCR

A qRT-PCR was performed to analyse the relative expression levels of viral mRNAs in GaHV2-infected CEFs. Briefly, total RNAs were extracted from GX0101 or mutant-infected CEFs, using the TRIzol reagent according to the manufacturer’s instructions (Invitrogen). To obtain cDNA, aliquots of 100 ng RNA were first treated with RNase-free DNase I (TaKaRa) and then polyadenylated and reverse transcribed at 37 °C for 1 h in a 20 µl reaction mixture, using the NCode VILO miRNA cDNA Synthesis Kit (Invitrogen) according to the manufacturer’s instructions. As described previously [38], the relative quantification of GaHV2 *meq*, *RLORF6*, *RLORF5a*, *RLORF4*, *gB* and *pp38* and chicken *β-actin* and *GAPDH* genes was performed using the primer pairs listed in Table S7 (primer pairs 9–16) and a 7500 Fast Real-Time PCR System (Applied Biosystems, Life Technologies). Relative quantification of the target gene expression was calculated with the 2^−△△Ct^ method. Each reaction was performed in triplicate repeats, and the data were calculated as mean±sd as described above.

## Supplementary Data

1182 Supplementary File 1
