# Supplementary material for: Putative roles as oncogene or tumour suppressor of the Mid-clustered microRNAs in Gallid alphaherpesvirus 2 (GaHV2) induced Marek’s disease lymphomagenesis
Source: J Gen Virol. 2017 May 11;98(5):1097–112. doi: 10.1099/jgv.0.000786 (PMC5656797; doi:10.1099/jgv.0.000786)
Supplement: Supplementary File 1 [file jgv-98-1097-s001.pdf]

Supplementary Tables

Table S1. Primers used for the construction and verification of mutated MDV viruses with deletions of the Mid-clustered miRNAs.

No.	Primer	Usage[a]	Type	Sequence (5’-3’)[b]	Length (nt)	Genomic binding site[c]	Amplicon (bp)[d]	
							GX0101	GXΔmiR
1	MidmiRF-Kan^R	Kan^R cassette	5’	GAAAGGGTTAAAGGCATTATTTATCGATTTACTGACAT<br>AAAAAAATCCTCCGTGTAGGCTGGAGCTGCTTC	71	134727-134777	NA	NA
	MidmiRR-Kan^R		3’	GGAGATTATCCCGAGAATTCAAACTATTCTTGTAATGT<br>CGTACGAGCCTCCATTCCGGGGATCCGTCGAC	70	135724-135675		
2	miR1F-Kan^R	Kan^R cassette	5’	GAGTAATCTGCGTTAAGTCGTTACTGGATTGTAACGG<br>CTATCCGGAGACTCGTGTAGGCTGGAGCTGCTTC	71	135545-135594	NA	NA
	miR1R-Kan^R		3’	GGAGATTATCCCGAGAATTCAAACTATTCTTGTAATGT<br>CGTACGAGCCTCCATTCCGGGGATCCGTCGAC	70	135724-135675		
3	miR11F-Kan^R	Kan^R cassette	5’	GAAAGGGTTAAAGGCATTATTTATCGATTTACTGACAT<br>AAAAAAATCCTCCGTGTAGGCTGGAGCTGCTTC	71	134727-134777	NA	NA
	miR11R-Kan^R		3’	CAGAAGTTTTCCACATAGCTAAGTTTATCTCATACTTCG<br>GAACTCCTGGACATTCCGGGGATCCGTCGAC	70	134906-134857		
4	miR31F-Kan^R	Kan^R cassette	5’	TAAGGAACAACACGATTGATCTCTCATCCGCGTCCCA<br>GCAATCAGGCCTACGTGTAGGCTGGAGCTGCTTC	71	135215-135264	NA	NA
	miR31R-Kan^R		3’	TTGCATAATTCATAAACGAATATTTAATTACAGTAGTGT<br>TGCGCGAGCCCCATTCCGGGGATCCGTCGAC	70	135394-135345		
5	Mid5F-135890	pmiR	5’	TCCGCATTGTGACTCTCAGCAG	22	134626-134647	NP	1517/284
	miR1R-Kan^R		3’	GGAGATTATCCCGAGAATTCAAACTATTCTTGTAATGT<br>CGTACGAGCCTCCATTCCGGGGATCCGTCGAC	70	135724-135675		
6	MDV_miR11-F	premiR	5’	GCCGAATTCAAATCCTCTGGGGTAACAA	28	134769-134787	958	1402/180
	MDV_miR1-R		3’	TCCCGAGAATTCAAACT	17	135700-135716		
7	Mid5F-135890	pmiR	5’	TCCGCATTGTGACTCTCAGCAG	22	134626-134647	NP	2337/1102

	miR1R-Kan^{R}		3’	GGAGATTATCCCGAGAATTCAAACTATTCTTGTAATGT	70	135724-135675		
				CGTACGAGCCTCCATTCCGGGGATCCGTCGAC				
8	MDV_miR1-F	premiR	5’	GGGGAATTCTGGATTGTAACGGCTAT	26	135569-135585	157	1383/161
	MDV_miR1-R		3’	TCCCGAGAATTCAAACT	17	135700-135716		
9	Mid5F-135890	pmiR	5’	TCCGCATTGTGACTCTCAGCAG	22	134626-134647	NP	1517/285
	miR11R-Kan^{R}		3’	CAGAAGTTTTCCACATAGCTAAGTTTATCTCATACTTCG	70	134906-134857		
				GAACTCCTGGACATTCCGGGGATCCGTCGAC				
10	MDV_miR11-F	premiR	5’	GCCGAATTCAAATCCTCTGGGGTAACAA	28	134769-134787	126	1390/130
	MDV_miR11-R		3’	CCCCTCGAGCATACTTCGGAACTCCTGGA	29	134857-134876		
11	Mid5F-135890	pmiR	5’	TCCGCATTGTGACTCTCAGCAG	22	134626-134647	NP	2007/773
	miR31R-Kan^{R}		3’	TTGCATAATTCATAAACGAATATTTAATTACAGTAGTGT	70	135394-135345		
				TGCGCGAGCCCCATTCCGGGGATCCGTCGAC				
12	MDV_miR31-F	premiR	5’	GCGGAATTCGTCCCAGCAATCAGGCCTAT	29	135246-135265	137	1373/141
	MDV_miR31-R		3’	CCCCTCGAGCAGTAGTGTTGCGCGAGCCC	29	135345-135364		
13	meq-Forward	meq	5’	ATGTCTCAGGAGCCAGAG	18	133603-133620	1020	1020
	meq-Reverse		3’	TCAGGGTCTCCCGTCACC	18	134605-134622		
14	pp38-Forward	pp38	5’	AATGTCGACACCGCACGCTTTGCTC	25	126556-126571	981	981
	pp38-Reverse		3’	ACAGGATCCTTAATCTCCGCCTCCAAC	27	125609-125626		
15	gB-Forward	gB	5’	ATGCACTATTTTAGGCGG	18	62365-62384	2296	2296
	gB-Reverse		3’	GGGCTTTCATTGGATTGC	18	60087-60104		
16	Kan5F	Kan^{R}	5’	CGTGTAGGCTGGAGCTGCTTC	21	NA	NA	NA
	Kan3R		3’	CATTCCGGGGATCCGTCGAC	20			
17	Ovo-Forward	OVO	5’	TTGAGTGGGAAGGCATAGAATC	22	NA	NA	NA
	Ovo-Reverse		3’	CGTCTCCTTTTCCGTCTTTCAA	22			
18	yg-meq-F	qPCR	5’	GTCCCCCCTCGATCTTTCTC	20	133656-133675	183	183
	yg-meq-R		3’	CGTCTGCTTCCTGCGTCTTC	20	133820-133839		
19	yg-gB-F	qPCR	5’	TCTAGGGCATGGCACACGAC	20	61835-61854	124	124

	yg-gB-R		3’	GAATACGGAAACACAGAGCGG	21	61730-61750		
20	yg-OVO-F	qPCR	5’	AAGCAAGAGAAATGGGCTGAT	21	NA	NA	NA
	yg-OVO-R		3’	AGGAGGGGAAGACATCCAGTA	21			

[a] Kan^R, Kanamycin resistance gene; pri-miR, primary miRNA; pre-miR, miRNA precursor; OVO, chicken ovotransferrin gene.

[b] Underlined sequences indicate the primers used to amplify the Kan^R gene cassette with FRT, and the other sequences indicate MDV sequence flanking the miRNA genes.

[c] Nucleotide numbering corresponds to the viral genome sequence of vvMDV strain GX0101 (GenBank Acc. No. JX844666).

[d] NA, not applicable; NP, no PCR product.

Table S2. Sequences of the PCR amplified regions of the partial primary miRNAs (primiR) from miRNA-deleted BAC clones or rescued viral genomes.

BAC/Strain	Primer pair [a]	Type	Sequence (5'-3') [b c]	Amplicon (bp)
**GXΔMid-miRs**	Mid5F-135890<br>miR1R-Kana^R	5'<br>3'	**TCCGCATTGTGACTCTCAGCAG**CACATCGTCTATGCCCCATGTTTCTTCTCCCCTAGTTATATATAATAGTTTTCATAGTTTCG<br>GGAAGATCAACATAAAGGAAAGGGTTAAAGGCATTATTTATCGATTTACTGACATAAAAAAATCCTC*CGTGTAGGCTGGAGCTG*<br>*CTTCGAAGTTCCTATACTTTCTAGAGAATAGGAACTTCGAACTGCAG**GTCGACGGATCCCCGGAATG***GAGGCTCGTACGACATT**<br>**ACAAGAATAGTTTGAATTCTCGGGATAATCTC**	284
**GXΔmiR-M11**	Mid5F-135890<br>miR11R-Kana^R	5'<br>3'	**TCCGCATTGTGACTCTCAGCAG**CACATCGTCTATGCCCCATGTTTCTTCTCCCCTAGTTATATATAATAGTTTTCATAGTTTCG<br>GGAAGATCAACATAAAGGAAAGGGTTAAAGGCATTATTTATCGATTTACTGACATAAAAAAATCCTC*CGTGTAGGCTGGAGCTG*<br>*CTTCGAAGTTCCTATACTTTCTAGAGAATAGGAACTTCGAACTGCAG**GTCGACGGATCCCCGGAATG***TCCAGGAGTTCCGAAGT**<br>**ATGAGATAAACTTAGCTATGTGGAAAACTTCTG**	285
**GXΔmiR-M31**	Mid5F-135890<br>miR31R-Kan^R	5'<br>3'	**TCCGCATTGTGACTCTCAGCAG**CACATCGTCTATGCCCCATGTTTCTTCTCCCCTAGTTATATATAATAGTTTTCATAGTTTCG<br>GGAAGATCAACATAAAGGAAAGGGTTAAAGGCATTATTTATCGATTTACTGACATAAAAAAATCCTCTGGGGTAACAAA*TTTTC*<br>*CTTACCGTGTAGCTTAGA*CTCGGAAGAACTATTT*TGAGTTACATGGTCAGGGGATT*TGTTGGCTCCAGGAGTTCCGAAGTATGA<br>GATAAACTTAGCTATGTGGAAAACTTCTGGGGCAACATCTCTCGGCCCCAGACTGCTTAAATGGCAAATTCTCGTTCTATACAG<br>AACGGTTGGGGAAGGGGAGGGGGGGGTATATGGAGTATTATTCGGGATATGGCTTCTATGAAGCCTGCGGTAAGTTTTCCAGGC<br>TCAAAAACTATGCCTGGCTGTTTTTTTTAGAAGGGATATGGACATCGCACATTAAGGAATATTAAAGATAACAGGATGGACATT<br>CGGATGTAAAAGGAATAAGCGAAACCTTTAGCAGATGTGAGTTAATGCAGTCTCGTATAATTCGGTGGTGCTGATTAGGTTATC<br>GTAAGGAACAACACGATTGATCTCTCATCCGCGTCCCAGCAATCAGGCCTA*CGTGTAGGCTGGAGCTGCTTCGAAGTTCCTATA*<br>*CTTCCTAGAGAATAGGAACTTCGAACTGCAG**GTCGACGGATCCCCGGAATG***GGGCTCGCGCAACACTACTGTAATTAAATATT**<br>**CGTTTATGAATTATGCAA**	773
**GXΔmiR-M1**	Mid5F-135890<br>miR1R-Kana^R	5'<br>3'	**TCCGCATTGTGACTCTCAGCAG**CACATCGTCTATGCCCCATGTTTCTTCTCCCCTAGTTATATATAATAGTTTTCATAGTTTCG<br>GGAAGATCAACATAAAGGAAAGGGTTAAAGGCATTATTTATCGATTTACTGACATAAAAAAATCCTCTGGGGTAACAAA*TTTTC*<br>*CTTACCGTGTAGCTTAGA*CTCGGAAGAACTATTT*TGAGTTACATGGTCAGGGGATT*TGTTGGCTCCAGGAGTTCCGAAGTATGA<br>GATAAACTTAGCTATGTGGAAAACTTCTGGGGCAACATCTCTCGGCCCCAGACTGCTTAAATGGCAAATTCTCGTTCTATACAG<br>AACGGTTGGGGAAGGGGAGGGGGGGGTATATGGAGTATTATTCGGGATATGGCTTCTATGAAGCCTGCGGTAAGTTTTCCAGGC	1102

	TCAAAAACTATGCCTGGCTGTTTTTTTTAGAAGGGATATGGACATCGCACATTAAGGAATATTAAAGATAACAGGATGGACATT<br>CGGATGTAAAAGGAATAAGCGAAACCTTTAGCAGATGTGAGTTAATGCAGTCTCGTATAATTCGGTGGTGCTGATTAGGTTATC<br>GTAAGGAACAACACGATTGATCTCTCATCCGCGTCCCAGCAATCAGGCCTATGTCCCTCTCCTGTGGCCAGCTCACTGGCTGTG<br>CACTGTGCGATTCTAAG*TGCTACAGTCGTGAGCAGATCAA*TGGATCGGGGCTCGCGCAACACTACTGTAATTAAATATTCGTTT<br>ATGAATTATGCAAATATGCACAGATAATATATACAGGGATGCACAGACATACTCCTATGCACCGATACACAGGCACATAGGCAG<br>ATGTCGACATTAACGAATATACAGGCACGGACCTCCAGGAACATATGGAAAATACCTCATCGCAGAGACGCTTATGCAGGAGTA<br>ATCTGCGGTTAAGTCGTTACTGGATTGTAACGGCTATCCGGAGACT*CGTGTAGGCTGGAGCTGCTTCGAAGTTCCTATACTTTC*<br>*TAGAGAATAGGAACTTCGAACTGCAG****GTCGACGGATCCCCGGAATG*****GAGGCTCGTACGACATTACAAGAATAGTTTGAATTCT**<br>**CGGGAATCTCC**

[a] Sequences and genomic loci of the presently used primers (underlined bold letters) are listed in Table S1. Kan^R, Kanamycin resistance gene.

[b] Sequences of corresponding regions amplified from the 'GXΔMid' or 'GXΔmiR' constructs, containing the sequences of the FRT sites (red italic letters) left with its flanking fragments (grey background) from Kan^R cassettes.

[c] Sequences of corresponding regions amplified from the 'GXΔMid' or 'GXΔmiR' constructs, containing the sequences of Kan^R cassettes are not shown.

Table S3. Procedure and number of birds used for sample collection or calculating the cumulative mortality and gross tumour occurrence in MDV-challenged chickens.

Group	Birds sacrificed for sample collection at different time points							Total	Early death		Total	Remained birds for calculating the mortality and gross tumour occurrence
	7 dpi	10 dpi	14 dpi	21 dpi	30 dpi	45 dpi	60 dpi		7 dpi	14 dpi		
**GX0101**	6	3	6	6	6	3	6	**36**	3	3	**6**	76-36-6=**34**
**GXΔmiR-M31**	6	0	6	6	6	3	6	**33**	1	3	**4**	76-33-4=**39**
**GXΔmiR-M11**	6	0	6	6	6	3	6	**33**	2	2	**4**	76-33-4=**39**
**GXΔmiR-M1**	6	0	6	6	6	3	6	**33**	0	1	**1**	76-33-1=**42**
**GXΔMid-miRs**	6	0	6	6	6	3	6	**33**	0	2	**2**	76-33-2=**41**
**Mock CEFs**	6	3	6	6	6	3	6	**36**	0	0	**0**	76-36-0=**40**

Table S4. The significance of difference on mortality and gross tumour occurrence in birds compared to each of the virus groups.

Group	Significance of difference on mortality [a]					Significance of difference on gross tumour occurrence [a]				
	GX0101	GXΔmiR-M31	GXΔmiR-M11	GXΔmiR-M1	GXΔMid-miRs	GX0101	GXΔmiR-M31	GXΔmiR-M11	GXΔmiR-M1	GXΔMid-miRs
GX0101	-	-	-	-	**	-	-	-	-	*
GXΔmiR-M31	-	-	-	*	**	-	-	-	-	*
GXΔmiR-M11	-	-	-	*	**	-	-	-	-	*
GXΔmiR-M1	-	*	*	-	*	-	-	-	-	-
GXΔMid-miRs	**	**	**	*	-	*	*	*	-	-

[a] Except for the birds sacrificed at 7, 10, 14, 21, 30, 45 and 60 dpi for sample collections and early deaths occurred before 14 dpi possibly due to intraperitoneal infection, totals of 34, 39, 39, 42 and 41 birds remained in groups of GX0101, GXΔmiR-M31, GXΔmiR-M11, GXΔmiR-M1 and GXΔMid-miRs were observed for calculating the mortality and gross tumour occurrence, respectively.

** Significant difference (p<0.01).

* Significant difference (p<0.05).

Table S5. Cumulative gross tumour occurrence in MDV-challenged chickens calculated at different days post-infection (dpi).

Time point (dpi)	Category	MDV strains & mock group [a]					
		GX0101 (55)	GXΔmiR-M31 (60)	GXΔmiR-M11 (60)	GXΔmiR-M1 (63)	GXΔMid-miRs (62)	Mock CEFs (61)
15	Gross tumours	-	-	-	-	-	-
	Tumour incidence	-	-	-	-	-	-
30	Gross tumours	11	4	9	10	10	-
	Tumour incidence	20.0%	6.7%	15.0%	15.9%	16.1%	-
45	Gross tumours	12	5	15	10	12	-
	Tumour incidence	21.8%	8.3%	25.0%	15.9%	19.4%	-
60	Gross tumours	14	7	17	12	13	-
	Tumour incidence	25.5%	11.7%	28.3%	19.0%	21.0%	-
75	Gross tumours	16	8	19	13	13	-
	Tumour incidence	29.1%	13.3%	31.7%	20.6%	21.0%	-
90	Gross tumours	22	19	27	20	/	-
	Tumour incidence	40.0%	31.7%	45.0%	31.7%	/	-

[a] Except for the birds sacrificed at 7, 10 and 14 dpi for sample collections and early deaths occurred before 14 dpi possibly due to intraperitoneal infection, totals of 55, 60, 60, 63, 62 and 61 birds remained in groups of GX0101, GXΔmiR-M31, GXΔmiR-M11, GXΔmiR-M1, GXΔMid-miRs and mock CEFs were observed for calculating the gross tumour occurrence, respectively.

"-" means that no gross tumours caused by virus infection were observed at corresponding time points post-challenge.

"/" means that there is no data applicable due to the death of all GXΔMid-miRs-challenged chickens before 75 dpi.

Table S6 Binding sites in the predicted viral mRNA targets of the Mid-clustered miRNAs.

No.	miRNA	Target candidates				mRNA/miRNA binding site	
		Gene	Product	Function	Gene ID [a]	Seed match	Location[b]
1	miR-M1-5p	MDV025	Tegument serine/threonine protein kinase	Protein phosphorylation	4811486	5’-AUGAAAUAACUCCCGAACAAGCAA-3’<br>|||||||| 3’- ACGGCGUGUCACUUGUUCGU -5’	3'-UTR
		MDV060	Tegument protein VP13/14	Possibly gene regulation	4811521	5’-CAUUACAGAAUAAUCAACAAGCGA-3’<br>||||||| 3’- ACGGCGUGUCACUUGUUCGU -5’	3'-UTR
2	miR-M11-5p	MDV076	Oncoprotein MEQ	Transformation	4811549	5’-GGGAAGAUCAACAUAAAGGAAAGG-3’<br>||||||| 3’-AGAUUCGAUGUGCCAUUCCUUUU-5’	3'-UTR
						5’- AUGAACACCUACGUAAGGAAAU-3’<br>|||||||| 3’-AGAUUCGAUGUGCCAUUCCUUUU -5’	CDS
						5’-UUCGAGACGGAAAAAAAGGAAAAG-3’<br>||||||| 3’- AGAUUCGAUGUGCAUUCCUUUU-5’	CDS

[a] Gene ID, annotated in the viral genome of Md5 (GenBank Acc. No. AF243438).

[b] 3'-UTR, 3'-untranslated region; CDS, coding domain sequence.

Table S7 Primers used for the construction of plasmid vectors for DLRA and qRT-PCR assays.

No.	Primer	Uasage [a]	Sequence (5'-3') [b,c]	Length (nt)	Amplicon (bp)
1	MDV-mir1-F	pre-miR	GGGGAATTCTGGATTGTAACGGCTAT	26	125
	MDV-mir1-R		TAACTCGAGCGTTCCGTTCGCTCTTT	26	
2	MDV-mir11-F	pre-miR	GCCGAATTCAAATCCTCTGGGGTAACAA	28	126
	MDV-mir11-R		CCCCTCGAGCATACTTCGGAACTCCTGGA	29	
3	MDV076tar(UTR)-F	3'-UTR	GCGCTCGAGGGTGTATACCAGGGAGAAGG	29	525
	MDV076tar(UTR)-R		ATAGCGGCCGCGGTGGAGGAGTGCAAAT	28	
4	MDV076tar(CDS)-F	CDS	GCGCTCGAGTCTACGGTCTGGTGGTTT	27	1008
	MDV076tar(CDS)-R		ATAGCGGCCGCCCAGTAACGACTTAACGC	29	
5	MDV025-M1-5p-F	3'-UTR	TCGAGCTTCGGAAGTCGTTTATGAAATAACTCCCGAACAAGCAATCCCTGTGC	53	53
	MDV025-M1-5p-R		GGCCGCACAGGGATTGCTTGTTCGGGAGTTATTTCATAAACGACTTCCGAAGC	53	
6	MDV060-M1-5p-F	3'-UTR	TCGAGACCAAGGCATACTCAAATTGGAGAAAGGCATGATACGAGGGC	47	47
	MDV060-M1-5p-R		GGCCGCCCTCGTATCATGCCTTTCTCCAATTTGAGTATGCCTTGGTC	47	
7	Mut-Meq-(UTR)-F	mut-3'-UTR	TCGAGCCATAGTTTCGGGAAGATCAACATA**TTCCTTTC**GGTTAAAGGC	48	48
	Mut-Meq-(UTR)-R		GGCCGCCTTTAACC**GAAAGGAA**TATGTTGATCTTCCCGAAACTATGGC	48	
8	Mut-M11-5p-F	mut-miR	AATTCG**AAACGCAA**CCGTGTAGCTTAGACTCGGAAGAACTATTTTGAGTTACATGGTCGCGGGTCTC	67	67
	Mut-M11-5p-R		TCGAGAGACCCGCGACCATGTAACTCAAAATAGTTCTTCCGAGTCTAAGCTACACGG**TTGCGTTT**CG	67	
9	qPCR-meq-F	qPCR	CGCAGGAAGCAGACGGACTA	20	157
	qPCR-meq-R		CCATAGGGCAAACTGGCTCAT	21	
10	qPCR-RLORF6-F	qPCR	AATGCGGATCATCAGGGTCTC	21	140
	qPCR-RLORF6-R		GAGAGGCTTTATGCTCGTCTTACC	24	
11	qPCR-RLORF5a-F	qPCR	AATACCTCATCGCAGAGACGC	21	162
	qPCR-RLORF5a-R		CTCGTTCCGTTCGCTCTTTC	20	
12	qPCR-RLORF4-F	qPCR	TGCTTGTTTTGGGTAATTGGTC	22	275

	qPCR-RLORF4-R		TACATTCCCCACGCTCACCAC	21	
13	qPCR-gB-F	qPCR	TCTAGGGCATGGCACACGAC	20	125
	qPCR-gB-R		GAATACGGAAACACAGAGCGG	21	
14	qPCR-pp38-F	qPCR	CCGAAAGACAAAACCCAAAT	20	129
	qPCR-pp38-R		ATGTAACCAGCATATAAGAACGC	23	
15	qPCR-β-actin-F	qPCR	GCACCACACTTTCTACAATGAGC	23	124
	qPCR-β-actin-R		GAAGGTCTCAAACATGATCTGTGTC	25	
16	qPCR-GAPDH-F	qPCR	AAGTCCCTGAAAATTGTCAGCAAT	24	119
	qPCR-GAPDH-R		ATGGCATGGACAGTGGTCATAAG	23	

[a] pre-miR, miRNA precursor; 3'-UTR, 3'-untranslated region; ORF, open reading frame; mut, mutation; qPCR, quantitative PCR.

[b] Restriction sites of *Eco*R I, *Xho* I and *Not* I are underlined.

[c] Mutated seed sequences of miRNA and target binding sites of 3’-UTRs are shown in bold.

[d] Universal-Check-R, universal reverse primer used for identification of recombinant psiCheck-2 vector.

[e] Universal-pcDNA-R, universal reverse primer used for identification of recombinant pcDNA6.2 vector.

Supplementary Figure Legends

Fig. S1. Schematics of the sequential steps for deletion of miR-M1 by BAC mutagenesis. Kan, kanamycin gene; CL, chloramphenicol gene; HA, 50-nt homology arms using for guiding the recombinations. The genomic loci are referenced to the viral genome of GaHV2 strain GX0101 (GenBank Acc. No. JX844666).

Fig. S2. Schematics of the sequential steps for deletion of miR-M11 by BAC mutagenesis. Kan, kanamycin gene; CL, chloramphenicol gene; HA, 50-nt homology arms using for guiding the recombinations. The genomic loci are referenced to the viral genome of GaHV2 strain GX0101 (GenBank Acc. No. JX844666).

Fig. S3. Schematics of the sequential steps for deletion of miR-M31 by BAC mutagenesis. Kan, kanamycin gene; CL, chloramphenicol gene; HA, 50-nt homology arms using for guiding the recombinations. The genomic loci are referenced to the viral genome of GaHV2 strain GX0101 (GenBank Acc. No. JX844666).

Figure S1

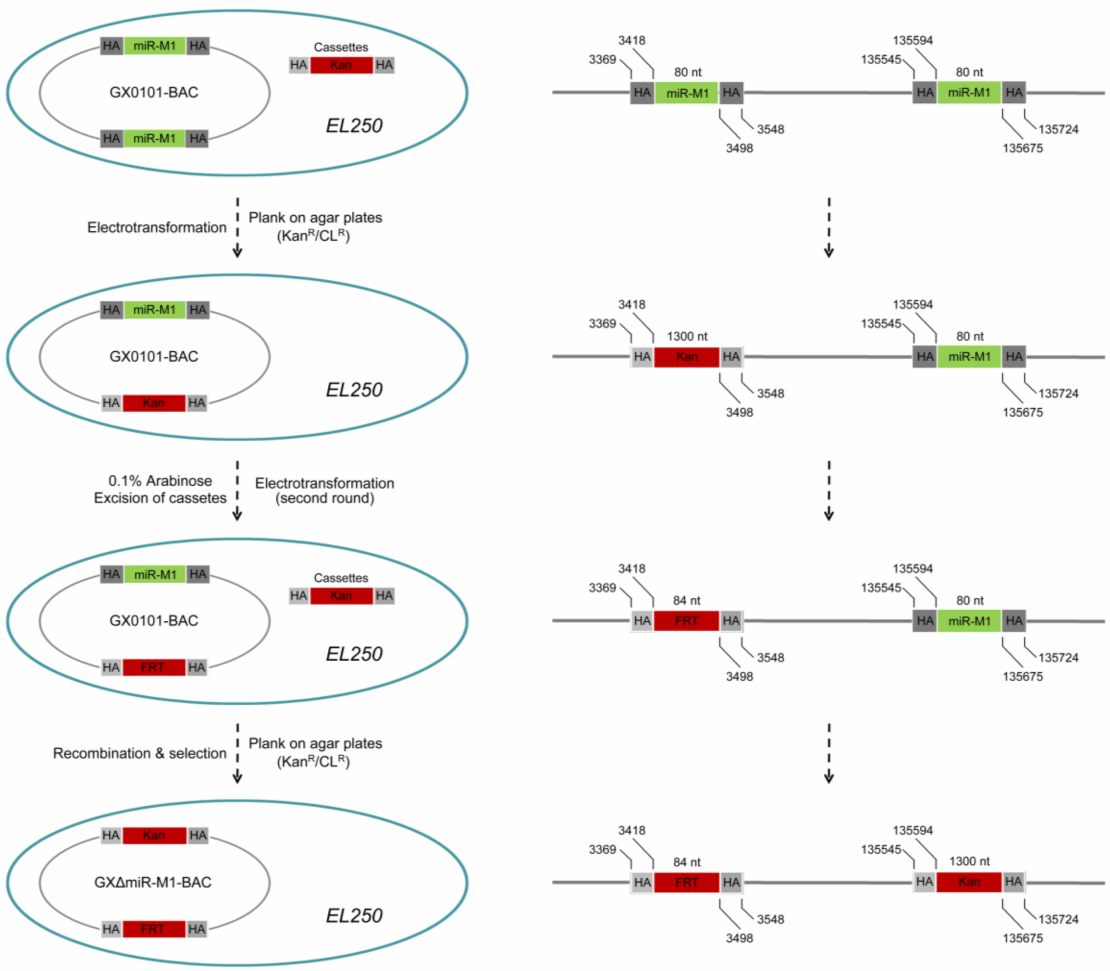
HA miR-M1 HA
Cassettes
HA Kan HA
GX0101-BAC
EL250
Electrotransformation
Plank on agar plates
(Kan^R/CL^R)
0.1% Arabinose
Excision of cassetes
Electrotransformation
(second round)
FRT
Recombination & selection
GXΔmiR-M1-BAC
3418
3369
80 nt
3548
3498
135594
135545
135724
135675
1300 nt
84 nt

Figure S2

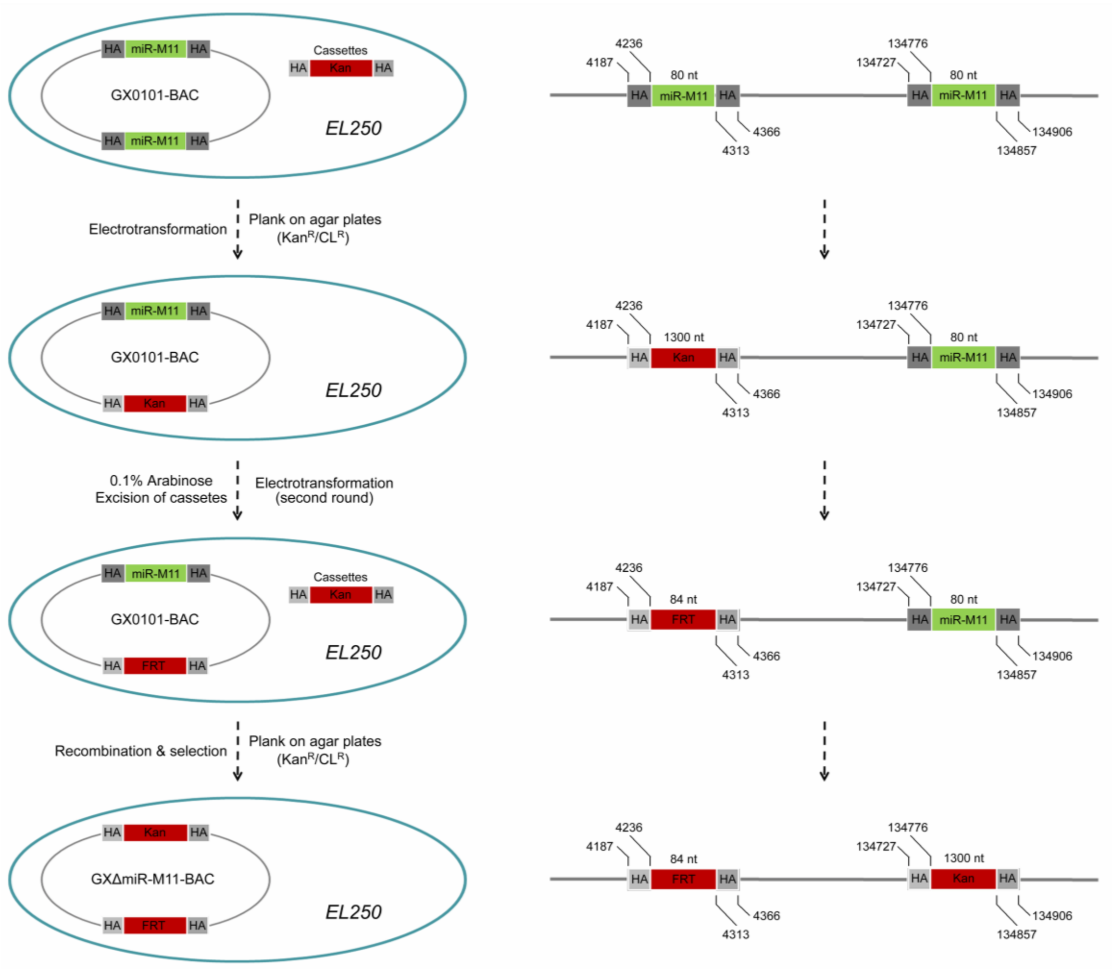

HA miR-M11 HA
Cassettes
HA Kan HA
GX0101-BAC
EL250
4187
4236
80 nt
4313
4366
134727
134776
134857
134906
Electrotransformation
Plank on agar plates
(KanR/CLR)
1300 nt
0.1% Arabinose
Excision of cassetes
Electrotransformation
(second round)
HA FRT HA
84 nt
Recombination & selection
GXΔmiR-M11-BAC

Figure S3

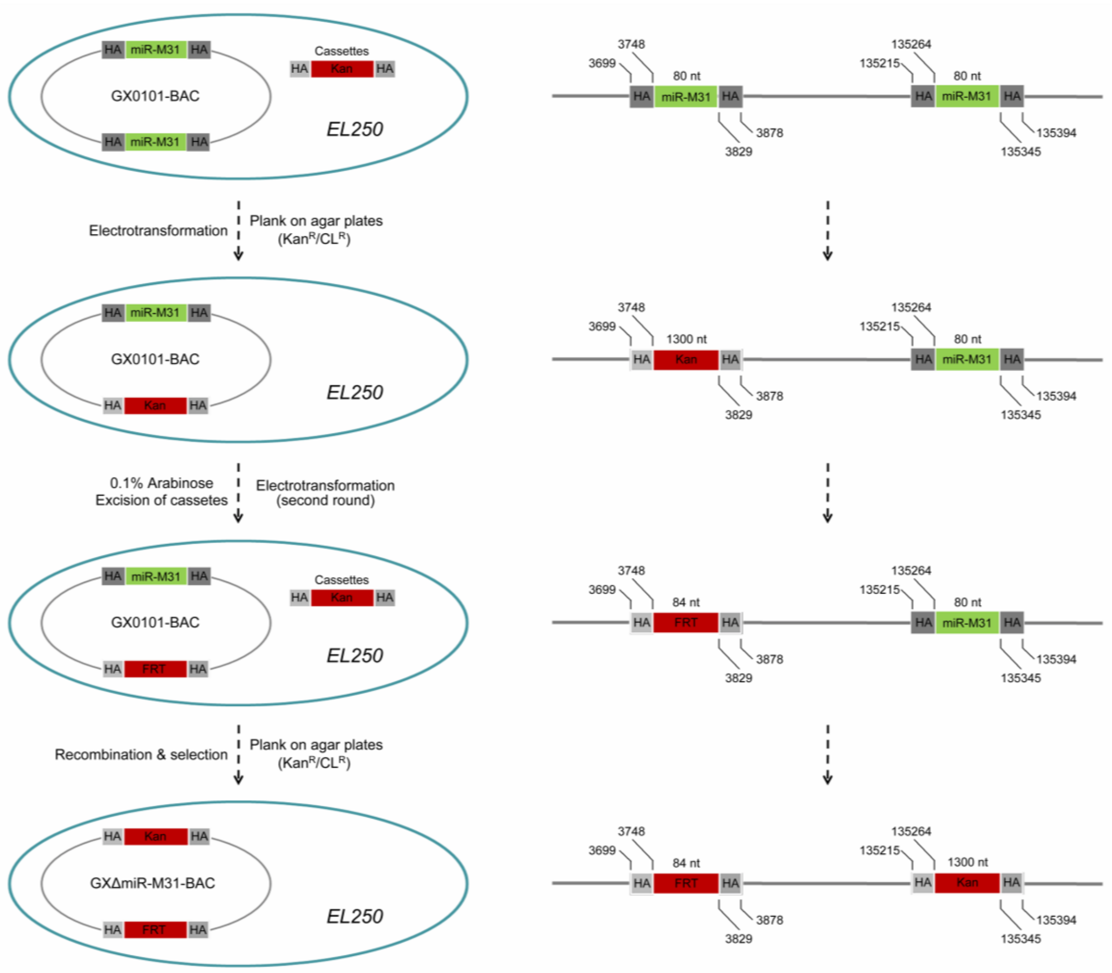
HA miR-M31 HA
Cassettes
HA Kan HA
GX0101-BAC
EL250
Electrotransformation
Plank on agar plates
(Kan^R/CL^R)
HA Kan HA
0.1% Arabinose
Excision of cassetes
Electrotransformation
(second round)
HA FRT HA
Recombination & selection
GXΔmiR-M31-BAC
3748
3699
80 nt
1300 nt
84 nt
3878
3829
135264
135215
135394
135345
